# Asthma exacerbations: a molecular dichotomy between antiviral and pro-inflammatory responses revealed

**DOI:** 10.1002/emmm.201202032

**Published:** 2012-11-14

**Authors:** Evangelos Andreakos

**Affiliations:** Center for Immunology and Transplantation, Division of Immunogenetics, Biomedical Research Foundation, Academy of AthensAthens, Greece

**Keywords:** allergic inflammation, asthma exacerbations, p65 NF-κB, rhinovirus, type I interferon

See related article in EMBO Molecular Medicine http://dx.doi.org/10.1002/emmm.201201650

Acute exacerbations are the major cause of morbidity, mortality and healthcare costs for individuals with asthma. They are the main cause of hospitalizations and are poorly controlled by current medication. It is therefore disappointing that we still know so little about the mechanistic details of asthma exacerbations. In this issue of *EMBO Molecular Medicine*, Bartlett et al. suggest that the transcription factor NF-κB, and specifically its subunit p65, is a key determinant of acute exacerbations of asthma, and propose a molecular dichotomy in the regulation of pro-inflammatory *versus* antiviral responses in asthmatic individuals that is mediated by p65 NF-κB and the type I interferon (IFN) system (Bartlett et al, 2012).

Asthma exacerbations are mostly triggered by respiratory infections of which rhinoviruses (RV), ssRNA viruses of the Picornaviridae family that cause common cold, are the most frequent (Busse et al, 2010). Other respiratory tract infections that can also trigger exacerbations include influenza, parainfluenza and respiratory syncytial virus in children. Asthmatic patients have increased susceptibility to respiratory infections, especially of the lower respiratory tract, and respiratory infections cause in turn more severe episodes of dyspnoea and wheezing in these patients. Presence of allergic sensitization further increases the risk for infections, the severity of exacerbations and the chances for hospital admission compared to non-sensitized asthmatic individuals. It is therefore essential to understand the complex interplay between sensitivity to infections, viral clearance and exacerbation of asthmatic inflammation in order to pave the way for the development of novel therapeutics.

…it is possible to dissociate antiviral from pro-inflammatory responses simply by inhibiting the p65 subunit of NF-κB…

To explain virus-induced exacerbations, several hypotheses have been advanced that attribute asthma attacks to immune-mediated exacerbations, direct viral injury, or both (Jackson et al, 2011). Virus infections can fuel innate and adaptive immune responses in the lung, eliciting neutrophilic cell infiltration, up-regulation of Th2-mediated responses in the airways and a new wave of inflammatory mediator release (cytokines, leukotrienes and histamine), altogether triggering bronchoconstriction and airway hyper-responsiveness. Viral infections can also compromise epithelial barrier integrity, increase goblet cell metaplasia, and alter neural mechanisms of bronchoconstriction and airway obstruction, directly exacerbating the disease. Central to both processes is defective viral clearance due to deficient IFN production and misdirected antiviral responses resulting in increased viral load, infections of the lower respiratory track and more severe episodes of asthma.

Up until now, induction or intensification of pro-inflammatory responses has been viewed as an effort of the organism to clear infection, and exacerbations of asthma have been considered as collateral damage that occurs as a result of this process. The fact that granulocytic cell infiltration, T-cell migration, cytokine production and leukotriene release are all essential components of antimicrobial immunity, while they also contribute to asthmatic exacerbations, has further supported this notion. The presumption has been that pro-inflammatory and antiviral responses are linked, and that common pathways are involved in their regulation. Bartlett et al now challenge this view. They suggest that it is possible to dissociate antiviral from pro-inflammatory responses simply by inhibiting the p65 subunit of NF-κB or by interfering with the type I IFN system. Using human bronchial epithelial cells, p65^+/−^ and IFN-αR^−/−^ mice, and a recently established mouse model of RV infections (Bartlett et al, 2008), the authors show that p65 NF-κB is essential for the exacerbation of allergic airway inflammation upon RV infection but is not required for the induction of type I/III IFNs, the infiltration of NK cells or T lymphocytes, or the clearance of RV from the lung. Rather, they show that these later events are dependent on type I IFN signalling, and suggest a dichotomy in the molecular pathways that control pro-inflammatory *versus* antiviral responses following RV infection (Fig 1).

**Figure 1:**
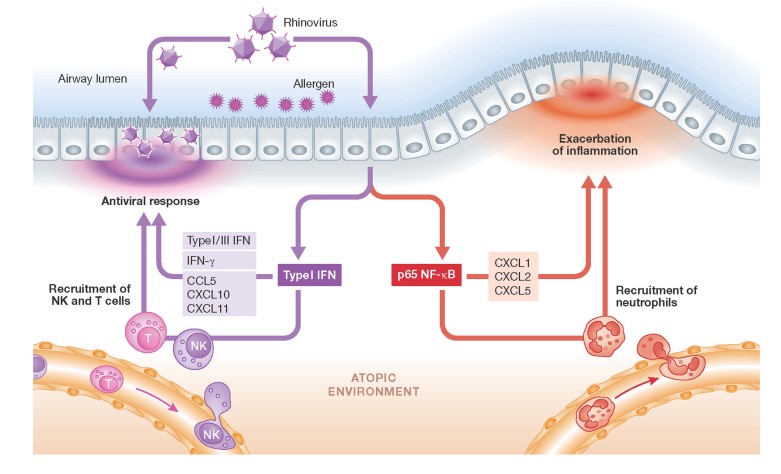
Type I IFN and p65 NF-κB define a molecular dichotomy between antiviral and pro-inflammatory responses during asthma exacerbations Rhinoviral infection of the bronchial epithelium triggers a p65 NF-κB-dependent/type I IFN-independent response that exacerbates airway inflammation and a type I IFN-dependent/p65 NF-κB-independent response that directs viral clearance.

The requirement for p65 NF-κB in the regulation of granulocytic cell infiltration does not come as a surprise. It has been previously shown that NF-κB regulates the expression of various neutrophil-recruiting chemokines such as CXCL1, CXCL2 and CXCL5, and controls the activation and persistence of neutrophils at sites of inflammation. What comes as a surprise is the lack of requirement for p65 in the induction of type I IFNs and the antiviral response. There is a strong body of evidence that NF-κB is essential for the formation of the transcriptional complex that drives the expression of type I IFNs (Apostolou & Thanos, 2008). The observation of Bartlett et al. that p65 NF-κB is redundant for the production of IFN-α, IFN-β and type III IFNs both *in vitro* and *in vivo* suggests that gene regulation in bronchial epithelial cells (which are also the most likely source of type I and III IFNs *in vivo*), and in response to rhinovirus, may differ. Indeed, it is becoming increasingly apparent that signalling pathways are dependent on the cell type, stimulus and context of activation, with rate-limiting steps in one setting being redundant in another (Andreakos et al, 2004). An explanation for this redundancy could be that different homo or heterodimeric NF-κB complexes lacking p65 are equally capable of binding to the promoters of type I and III IFNs and driving their expression (Balachandran & Beg, 2011). Still, the experiments of Bartlett et al. in bronchial epithelial cells with a non-degradable form of IκBα argue against this possibility.

Another unexpected yet exciting finding was the observation that p65 NF-κB is not required for the antiviral immune response and pathogen clearance but that this is rather mediated by the type I IFN system. As there is a large body of evidence that NF-κB is essential for the generation of adaptive immunity, the present study points to a rate-limiting role of the initial innate immune response in RV clearance. This appears to be orchestrated by type I IFNs that act coordinately to amplify their own expression, induce IFN-γ and type III IFNs, promote the release of chemokines and the recruitment of effector cells to the site of infection, and establish a localized antiviral state. The net effect is reduction of peak viral load and rapid viral clearance. The ability of type I IFNs to recruit NK and T cells to the lung in response to respiratory viral (RV) infection has not been suspected. The authors suggest that this is due to the up-regulation of key lymphocyte-recruiting chemokines such as CCL5, CXCL10 and CXCL11 as these are severely compromised in IFN-αβR^−/−^ mice, while there is scattered evidence in the literature that type I IFNs may control chemokine expression directly. Thus, although type I IFNs have been extensively studied for over three decades and multiple facets of these cytokines have been described including their role in the inhibition of viral replication, the activation of cytotoxic NK- and T-cell responses, the regulation of T-helper-cell function and the modulation of DC function (Gough et al, 2012), there is still more to be learnt.

This study still leaves a number of key questions unanswered. First, it does not explore in full the effect of RV infections in asthma exacerbations, for example, it does not provide information about T-helper-cell responses, airway remodelling or airway hyper-responsiveness. Second, it does not dissect the type I IFN-mediated mechanism of viral clearance nor the nature of the effector response. Third, it does not investigate the importance of type III IFNs, despite the fact that these cytokines share several of their antiviral activities with type I IFNs and exhibit potent immunomodulatory activity in allergic airway disease (Koltsida et al, 2011). Finally, it does not address the role of complete NF-κB inhibition in asthma exacerbations, an important aspect of this work as it will be very difficult, if not impossible, to develop specific p65 NF-κB inhibitors for the clinic. This study also has an important limitation. It is mostly based on a specific animal model of RV-induced asthma exacerbations and although this is the only *in vivo* model that can provide insight into the pathophysiological mechanisms of RV-induced exacerbations of asthma, it is still suboptimal in modelling the human situation. Nevertheless, this study is the first to show in an *in vivo* experimental setting that it is possible to dissect the ‘disease exacerbating’ pro-inflammatory responses from the ‘beneficial’ antiviral responses in asthma. From the translational perspective, this means that it may be possible to selectively suppress pro-inflammatory responses during exacerbations of asthma without compromising antiviral immunity or, alternatively, to boost antiviral immunity without exacerbating ‘asthmatic’ inflammation. This is therefore a major step forward towards the development of new strategies for the treatment of acute asthma exacerbations.

## References

[b1] Andreakos E, Sacre SM, Smith C, Lundberg A, Kiriakidis S, Stonehouse T, Monaco C, Feldmann M, Foxwell BM (2004). Distinct pathways of LPS-induced NF-kappa B activation and cytokine production in human myeloid and nonmyeloid cells defined by selective utilization of MyD88 and Mal/TIRAP. Blood.

[b2] Apostolou E, Thanos D (2008). Virus Infection Induces NF-kappaB-dependent interchromosomal associations mediating monoallelic IFN-beta gene expression. Cell.

[b3] Balachandran S, Beg AA (2011). Defining emerging roles for NF-kappaB in antivirus responses: revisiting the interferon-beta enhanceosome paradigm. PLoS Pathog.

[b4] Bartlett NW, Walton RP, Edwards MR, Aniscenko J, Caramori G, Zhu J, Glanville N, Choy KJ, Jourdan P, Burnet J (2008). Mouse models of rhinovirus-induced disease and exacerbation of allergic airway inflammation. Nat Med.

[b5] Bartlett NW, Slater L, Glanville N, Haas JJ, Caramori G, Casolari P, Clarke DL, Message SD, Aniscenko J, Kebadze T (2012). Defining critical roles for NF-κB p65 and type I interferon in innate immunity to rhinovirus. EMBO Mol Med.

[b6] Busse WW, Lemanske RF, Gern JE (2010). Role of viral respiratory infections in asthma and asthma exacerbations. Lancet.

[b7] Gough DJ, Messina NL, Clarke CJ, Johnstone RW, Levy DE (2012). Constitutive type I interferon modulates homeostatic balance through tonic signaling. Immunity.

[b8] Jackson DJ, Sykes A, Mallia P, Johnston SL (2011). Asthma exacerbations: origin, effect, and prevention. J Allergy Clin Immunol.

[b9] Koltsida O, Hausding M, Stavropoulos A, Koch S, Tzelepis G, Ubel C, Kotenko SV, Sideras P, Lehr HA, Tepe M (2011). IL-28A (IFN-lambda2) modulates lung DC function to promote Th1 immune skewing and suppress allergic airway disease. EMBO Mol Med.

